# Sensitive and reproducible cell-free methylome quantification with synthetic spike-in controls

**DOI:** 10.1016/j.crmeth.2022.100294

**Published:** 2022-09-09

**Authors:** Samantha L. Wilson, Shu Yi Shen, Lauren Harmon, Justin M. Burgener, Tim Triche, Scott V. Bratman, Daniel D. De Carvalho, Michael M. Hoffman

**Affiliations:** 1 Princess Margaret Cancer Centre, University Health Network, Toronto, ON, Canada; 2 Van Andel Institute, Grand Rapids, MI, USA; 3 Department of Medical Biophysics, University of Toronto, Toronto, ON, Canada; 4 Department of Computer Science, University of Toronto, Toronto, ON, Canada; 5 Vector Institute for Artificial Intelligence, Toronto, ON, Canada

**Keywords:** cell-free methylated DNA immunoprecipitation, cfMeDIP, spike-in controls, DNA methylation, cell-free DNA, cfDNA, liquid biopsy, absolute quantification, minimally invasive testing, reference standards, batch effects, early detection of cancer

## Abstract

Cell-free methylated DNA immunoprecipitation sequencing (cfMeDIP-seq) identifies genomic regions with DNA methylation, using a protocol adapted to work with low-input DNA samples and with cell-free DNA (cfDNA). We developed a set of synthetic spike-in DNA controls for cfMeDIP-seq to provide a simple and inexpensive reference for quantitative normalization. We designed 54 DNA fragments with combinations of methylation status (methylated and unmethylated), fragment length (80 bp, 160 bp, 320 bp), G + C content (35%, 50%, 65%), and fraction of CpG dinucleotides within the fragment (1/80 bp, 1/40 bp, 1/20 bp). Using 0.01 ng of spike-in controls enables training a generalized linear model that absolutely quantifies methylated cfDNA in MeDIP-seq experiments. It mitigates batch effects and corrects for biases in enrichment due to known biophysical properties of DNA fragments and other technical biases.

## Highlights

• Synthetic spike-in DNA provides a known reference for normalizing cfMeDIP-seq
• Spike-ins enable absolute quantification of cfDNA methylation at the picomole scale
• Spike-ins can adjust quantification for fragment length, G + C content, and CpG fraction
• Spike-ins reduce batch effects and batch-associated variance to <1% of total

## Motivation

For robust quantitative comparisons between samples, immunoprecipitation enrichment methods such as cfMeDIP-seq require normalization against common reference controls. Common reference controls can correct for technical variation in the processing of different samples. They can also correct for biases in enrichment due to known biophysical properties of DNA fragments such as fragment length, G + C content, and fraction of CpG dinucleotides. Furthermore, common reference controls can provide an experimental standard for quality control. We sought to provide synthetic spike-in DNA fragments that would fulfill these reference control purposes in cfMeDIP-seq experiments.

## Graphical abstract

**Figure:**
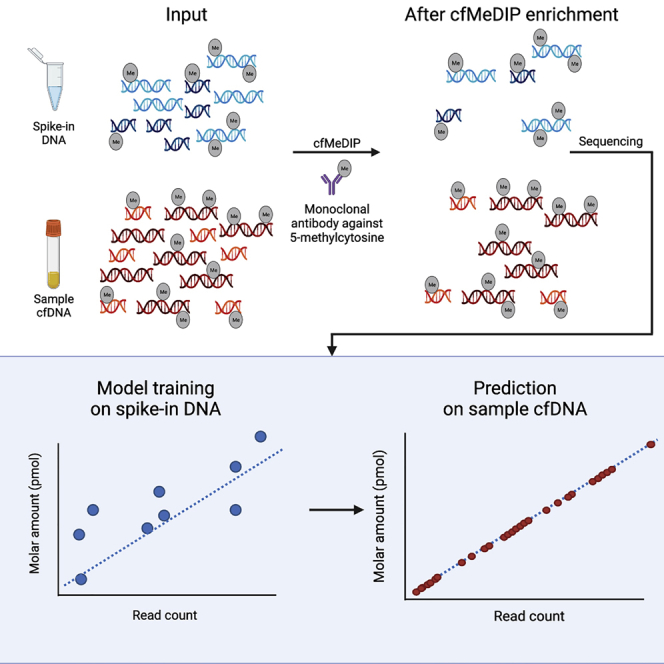

## Teaser

Wilson et al. developed synthetic DNA controls for normalizing cell-free methylation DNA immunoprecipitation sequencing (cfMeDIP-seq) assays used in liquid biopsies. These spike-in controls allow absolute quantification of methylated cell-free DNA in picomoles rather than arbitrary read counts. They also adjust for batch effects and reduce systematic noise related to technical biases.

## Introduction

Cell-free methylated DNA immunoprecipitation sequencing (cfMeDIP-seq) identifies DNA methylation using low-input samples of cell-free DNA (cfDNA). This method detects DNA methylation patterns reflective of distinct cancer types from circulating tumor DNA, which arises from tumor cells shedding DNA into an individual’s blood (Shen et al. 2018, 2019). cfMeDIP-seq proves ideal when assessing peripheral blood plasma from cancer patients, where one may obtain only a small amount of circulating tumor DNA and no indication of from where the circulating tumor DNA originates.

Sequencing assay methods, such as cfMeDIP-seq or RNA sequencing (RNA-seq), require a control for more accurate quantitative comparison across samples and batches. Reference controls for sequencing assays have consisted of spike-in reference DNA or RNA fragments of known sequence (Jiang et al., 2011; Chen et al., 2016; Orlando et al., 2014; Deveson et al., 2016; Blackburn et al., 2019). Without spike-in controls, one must assume that a given amount of assayed material produces equal DNA or RNA yields in different experimental conditions, and that this also holds true across all genomic regions (Chen et al., 2016). By normalizing quantification to a known amount of spike-in DNA added into a sample, we can overcome this assumption, leading to more accurate results (Chen et al., 2016). When carefully designed, spike-in controls can adjust for specific technical biases.

In addition to adjusting for technical biases, spike-in controls act as experimental standards for quality control. The addition of spike-in controls dramatically changes the interpretation of RNA-seq, chromatin immunoprecipitation sequencing, and other genomic assay results (Jiang et al., 2011; Chen et al., 2016; Orlando et al., 2014; Deveson et al., 2016; Blackburn et al., 2019). As such, all quantitative genome-wide assays would benefit from the addition of spike-in controls (Chen et al., 2016).

The most common approach to normalizing sequencing assay data consists in dividing the number of reads at each genomic region by the total number of reads genome-wide. This approach addresses technical variance owing to sequencing depth, but it can mask differences in biological variables of interest. Normalizing data to a known amount of spike-in DNA for each sample allows for more accurate detection of differences and adjustment of biophysical properties of DNA fragments that can influence results (Orlando et al., 2014; Chen et al., 2016).

While other genomic assays have long utilized spike-in controls, methods measuring genome-wide DNA methylation have rarely used them. The previous cfMeDIP-seq protocol uses methylated and unmethylated *Arabidopsis thaliana* DNA as a spike-in control to assess immunoprecipitation and binding efficiency to methylated DNA (Shen et al., 2019). This approach cannot correct for properties of specific methylated DNA fragments likely to influence results, such as G + C content, fragment length, and CpG fraction. Other controls used in DNA methylation enrichment methods include setting aside and sequencing a portion of input DNA without the enrichment procedure (Plongthongkum et al., 2014). This provides a reference point to assess enrichment of DNA methylation overall. Sequencing input DNA, however, cannot adjust for properties of individual fragments that can affect enrichment. Spike-in controls for bisulfite conversion methods for assaying DNA methylation (Liu et al., 2019; Olova et al., 2018) have no use in bisulfite-free enrichment methods, such as cfMeDIP.

Here, we introduce new synthetic DNA spike-in controls for cfMeDIP-seq. Our synthetic spike-ins can measure how DNA fragment properties, such as length, G + C content, and number of CpGs, can affect the number of reads produced by some known amount of fragment. Our spike-in controls assess non-specific binding, an integral part of cfMeDIP-seq analysis. The spike-in controls also mitigate technical effects such as experiments performed by different labs. We also use unique molecular indices (UMIs) to adjust for PCR bias. To calculate methylation specificity, we compare methylated fragments with unmethylated fragments after cfMeDIP-seq. We add a known molar amount of spike-in controls to each sample. With this information, we apply a generalized linear model to calculate molar amount, accounting for fragment length, G + C content, and CpG fraction. These spike-in controls generate an absolute quantitative measure of methylated DNA, allowing for more robust comparisons between samples and experiments.

## Results

### Spike-in controls confirm cfMeDIP’s high efficiency and specificity

**Figure 1:**
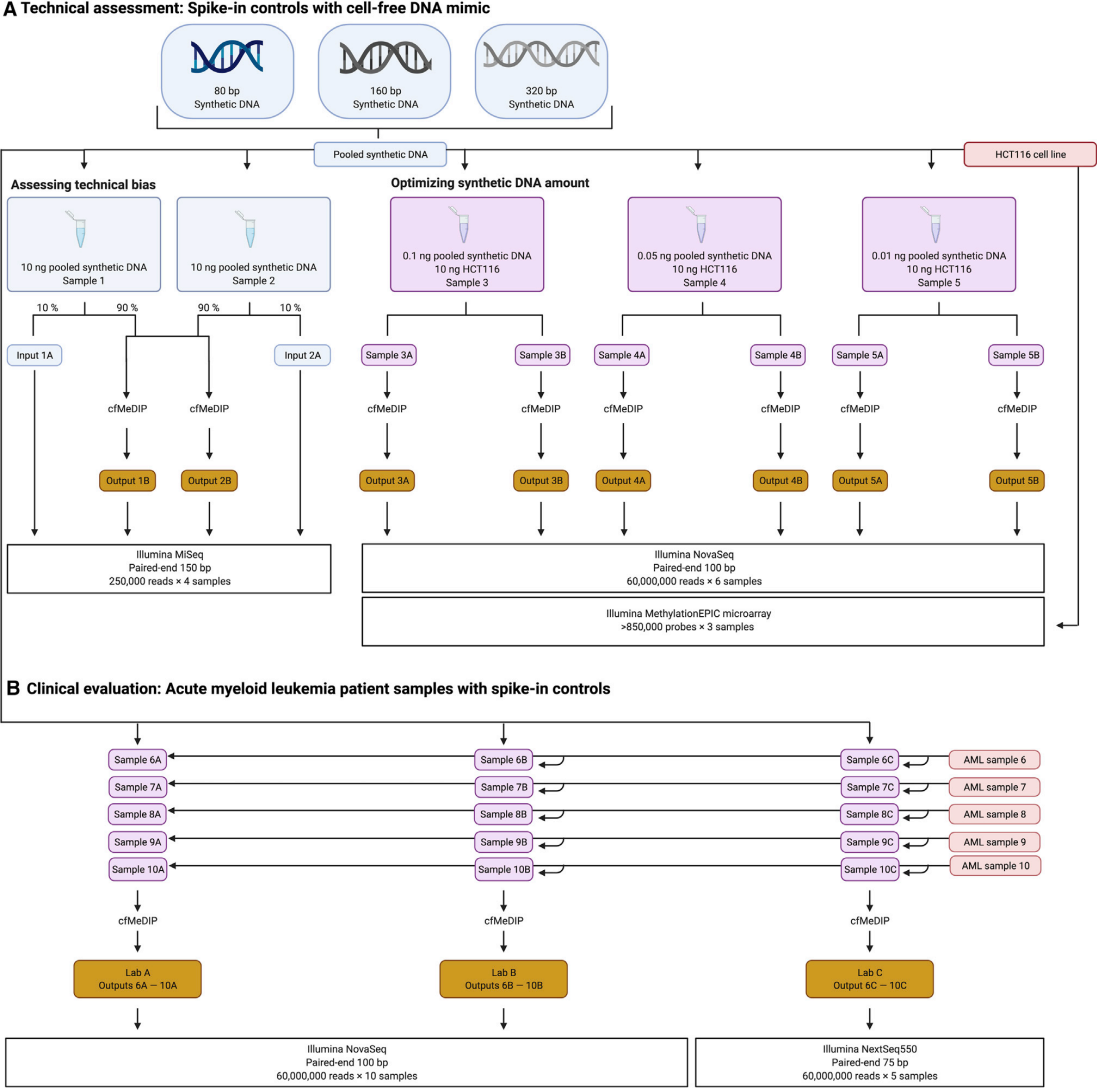
Experimental design using synthetic spike-in control DNA (A) Technical assessment of the spike-in controls with cfDNA mimic. (Left) Assessment of technical bias in solely the spike-in controls. (Right) Optimization of the synthetic DNA amount using sheared HCT116 cfDNA mimic. (B) Clinical evaluation of acute myeloid leukemia (AML) patient samples with spike-in controls.

**Figure 2:**
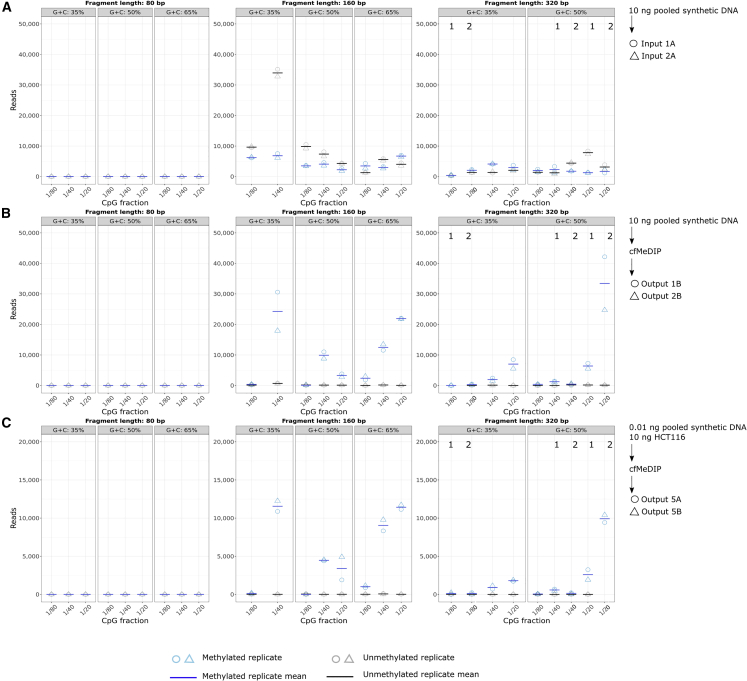
Assessing biases in fragment length, G + C content, and CpG fraction (A–C) In (A) input spike-in control DNA without cfMeDIP-seq, (B) output spike-in control DNA, after cfMeDIP-seq, and (C) 0.01 ng spike-in control DNA added to HCT116 replicates. Blue, methylated fragments; gray, unmethylated fragments. Circle, sample 1; triangle, sample 2. Solid line, mean of the two samples. Columns marked with numerals 1 and 2 represent alternative sets of fragments with identical properties but different sequences. See also Table S2.

We performed cfMeDIP-seq directly on the synthetic spike-in control fragments (Figure 1A, left; Table S1). For each sample, we saved 10% of the mass before performing cfMeDIP-seq. This acts as an input control. In input samples, we observed 51% of the input fragments methylated and 49% unmethylated. After cfMeDIP, 97% of the output fragments were methylated (Figures 2A and 2B; Table S2). The enrichment for methylated sequences further supports the validity and high efficiency of cfMeDIP-seq.

After cfMeDIP, signal from methylated fragments for both the synthetic spike-in control alone (97% of spike-in control fragments) and 10 ng of HCT116 colon cancer cell line DNA with 0.01 ng of spike-in (97% of spike-in control fragments) showed an enrichment of 160 bp fragments. We expected this enrichment, owing to our size selection step for insert size fragments between 80 bp and 380 bp. We designed fragments outside these insert sizes (1) to assess the efficiency of size selection and (2) for use in experiments without size selection. We also observed enrichment of fragments with higher G + C content and higher CpG fraction (Figures 2B and 2C; Table S2), and observed depletion in fragments with fewer CpGs. Immunoprecipitation less often enriches these fragments, methylated or unmethylated. Our spike-in controls allow us to account for this bias, facilitating the accurate interpretation of cfMeDIP-seq data.

Signal from unmethylated fragments for both the synthetic spike-in control alone (3% of spike-in control fragments) and 10 ng of HCT116 DNA with 0.01 ng of spike-in (3% of spike-in control fragments) had no association with fragment lengths, G + C content, or CpG fraction (Figures 2B and 2C; Table S2). This suggests that the low amount of unmethylated fragment signal arose from random non-specific binding and that it was not confounded systematically with fragment length, G + C content, or CpG fraction.

We also observed similar results in the acute myeloid leukemia (AML) samples. These samples had more unmethylated reads and lower methylation specificity in Lab C (Table S3). The lower methylation specificity arose from not using the methylated filler specified in the original protocol (Shen et al. 2018, 2019).

We assessed the range of number of sequenced reads where we had alternative sets of 320 bp fragments with different G + C contents and CpG fractions. Because no unmethylated fragment yielded more than 20 reads in any experiment, we focused our assessment on methylated fragments. The similar numbers of reads sequenced for alternative fragments with the same fragment properties show the robustness of the spike-in controls (Table 1).

**Table 1:** Range of reads sequenced for alternative synthetic spike-in fragments of length 320 bp with the same G + C contents and CpG fractions

Experiment	G + C content (%)	CpG fraction	Within-alternative reads^a^	Between-alternatives reads^b^
10 ng of synthetic DNA	35	1/80	0–343	343
	50	1/40	298–445	1,161
	50	1/20	1,759–17,472	36,642
HCT116 + 0.01 ng of synthetic DNA	35	1/80	13–219	219
	50	1/40	160–240	646
	50	1/20	987–1,320	8,483

a Minimum and maximum range between the individual replicates of two alternatives.

b Range between the means of two alternatives.

Even in the fragments with the largest range between alternatives (50% G + C content, 1/20 bp CpG fraction), this range represents only ≤4% of the total number of sequenced reads per sample. This difference may represent unknown fragment properties that influence cfMeDIP-seq results. The other sets of alternative fragments had even smaller ranges between alternatives.

### Low-input spike-in control accounts for technical variance

To determine the optimal amount of spike-in control DNA to add to each sample, we assessed the proportion of reads used toward our spike-in controls. We compared spike-in control reads with the total number of reads used for our biological sample, HCT116 genomic DNA (Figure 1A, right). We optimized the amount of spike-in controls to be used in subsequent experiments. This allowed us to maximize reads to our biological sample of interest while obtaining sufficient reads from the spike-in controls to correct for technical bias. Adding in 0.01 ng of our synthetic spike-in control DNA before cfMeDIP-seq used <1% of the reads for the spike-in controls. We retained >650,000 reads of spike-in control sequence for analysis while leaving the rest of the reads for our biological sample. Therefore, we decided to use 0.01 ng of spike-in control fragments in subsequent experiments.

The 0.01 ng of spike-in control DNA added to 10 ng of HCT116 genomic DNA revealed ≥97% specificity of methylated DNA with ≤0.01% total non-specific binding to non-methylated fragments (Figure 2C and Table S2). We calculated methylation specificity by dividing the total number of methylated fragments by the total number of spike-in control fragments. The cfMeDIP process also enriched for the 160 bp fragments we physically size selected for and for higher G + C content. Fragments that have CpG present at only 1/80 bp in the experiment with 10 ng of HCT116 with 0.01 ng of spike-in control DNA were represented by ≤1% of reads (Figure 2C and Table S2). The same patterns persisted when spiking in 0.05 ng and 0.1 ng of spike-in control DNA into 10 ng of HCT116 cell line.

### Removing problematic regions eliminates some technical artifacts

From our normalized data, we removed regions containing simple repeats, the Encyclopedia of DNA Elements (ENCODE) Project (ENCODE Project Consortium 2012) blacklist (Amemiya et al., 2019) regions, and regions with mappability scores of ≤0.5. We noticed that many regions with high molar amount also had high standard deviation of molar amount between replicate samples. Thus, we removed regions where standard deviation of molar amount between replicates was ≥0.05.

**Figure 3:**
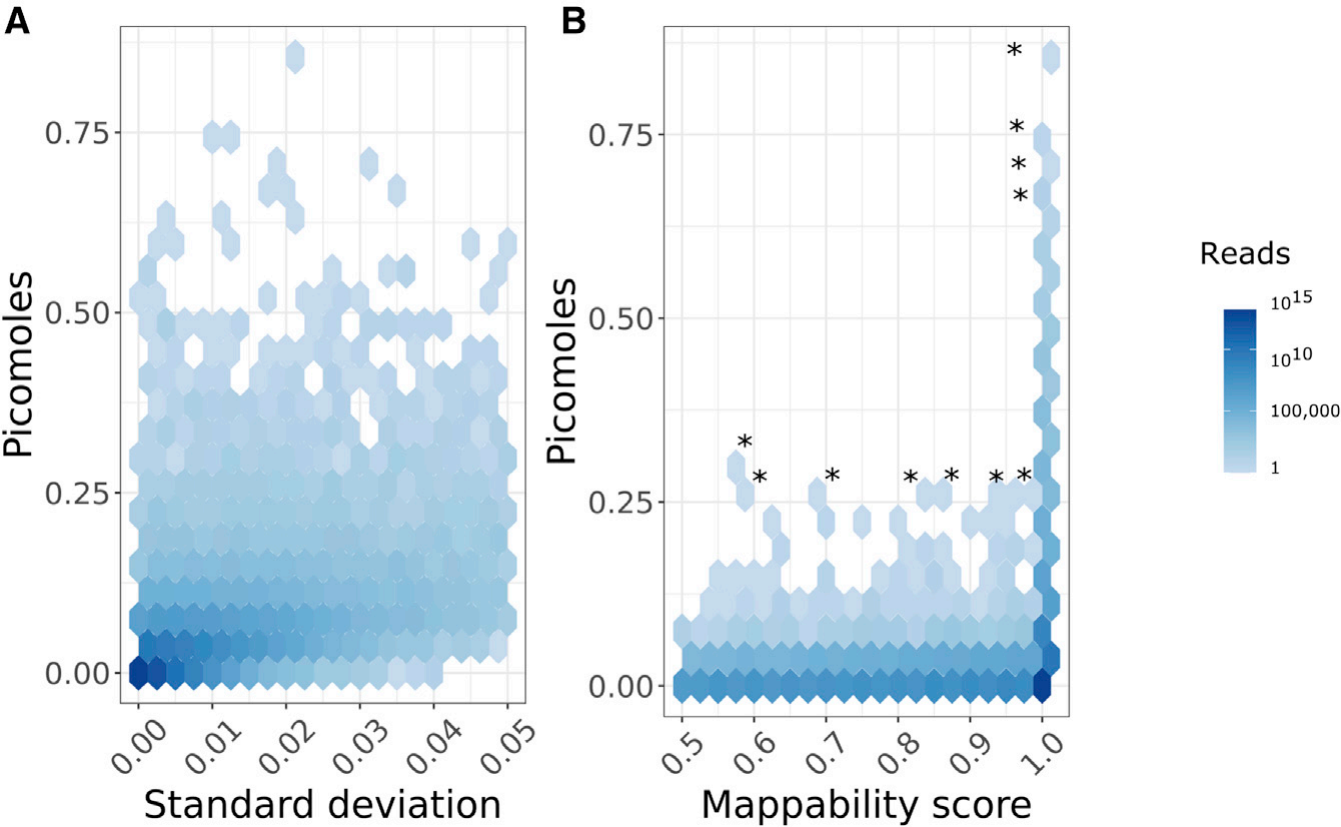
Two-dimensional histograms of the number of reads found in 300 bp windows (A and B) Binned by molar amount and either (A) standard deviation of molar amount or (B) Umap k100 multi-read mappability. Histograms only include windows that do not overlap with UCSC simple repeats and the ENCODE blacklist, and regions with Umap k100 multi-read mappability scores ≤0.5. Asterisks indicate 11 outlier genomic windows chosen for further examination.

After removing the high standard deviation regions, we observed no relationship between molar amount and standard deviation of molar amount, and no relationship between molar amount and mappability (Figure 3). We further examined the 11 genomic windows with the highest estimated molar amounts (≥0.25 pmol; Figures 3A and 3B). Of these 11 windows, seven overlapped repetitive elements (Table 2). Of those seven, six overlapped with Alu elements. This suggests that removing regions that have high standard deviation of molar amount between replicates can reduce technical artifacts. Regions with high standard deviation of molar amount between replicates perform inconsistently, leading to inaccurate quantification of DNA methylation.

**Table 2:** 11 genomic windows of length 300 bp with high predicted molar amount

Chr^a^	Start^b^	End^b^	Amount	Gene^c^	Repeat element^d^	Repeat family^d^	Repeat name^d^
**Windows with amount ≥0.70 pmol (n = 4)**							

19	308,701	309,000	0.86 pmol	MIER2	–	–	–
19	343,501	343,800	0.74 pmol	MIER2	–	–	–
19	613,201	613,500	0.73 pmol	HCN2	–	–	–
19	651,601	651,900	0.70 pmol	RNF126	–	–	–

**Windows with amount ≥0.25 pmol and mappability score <1 (n = 7)**							

6	495,301	495,600	0.31 pmol	EXOC2	SINE, LINE	Alu, L1	AluYk2, L1ME2z
6	426,601	426,900	0.28 pmol	–	LTR	ERV1	LTR12C
19	759,601	759,900	0.27 pmol	MISP	SINE	Alu	AluY
6	4,048,201	4,048,500	0.26 pmol	PRPF4B	SINE	Alu	AluY
19	671,401	671,700	0.25 pmol	–	SINE, SINE, SINE	Alu, Alu, Alu	AluSx4, AluYa8, MIRb
19	958,501	958,800	0.25 pmol	ARID3A	SINE	Alu	AluY
19	858,001	858,300	0.25 pmol	–	SINE	Alu	AluY

Sorted by decreasing molar amount.

a Chromosome.

b GRCh38/hg38, genomic position 1-start, fully closed.

c Symbols of GENCODE version 33 basic gene set genes (Frankish et al., 2019) that overlap our 300 bp genomic windows.

d Elements, families, and names of RepeatMasker (Smit et al., 2010) version 3.0 repeats that overlap our 300 bp genomic windows.

### Absolute quantification correlates with M-values

**Figure 4:**
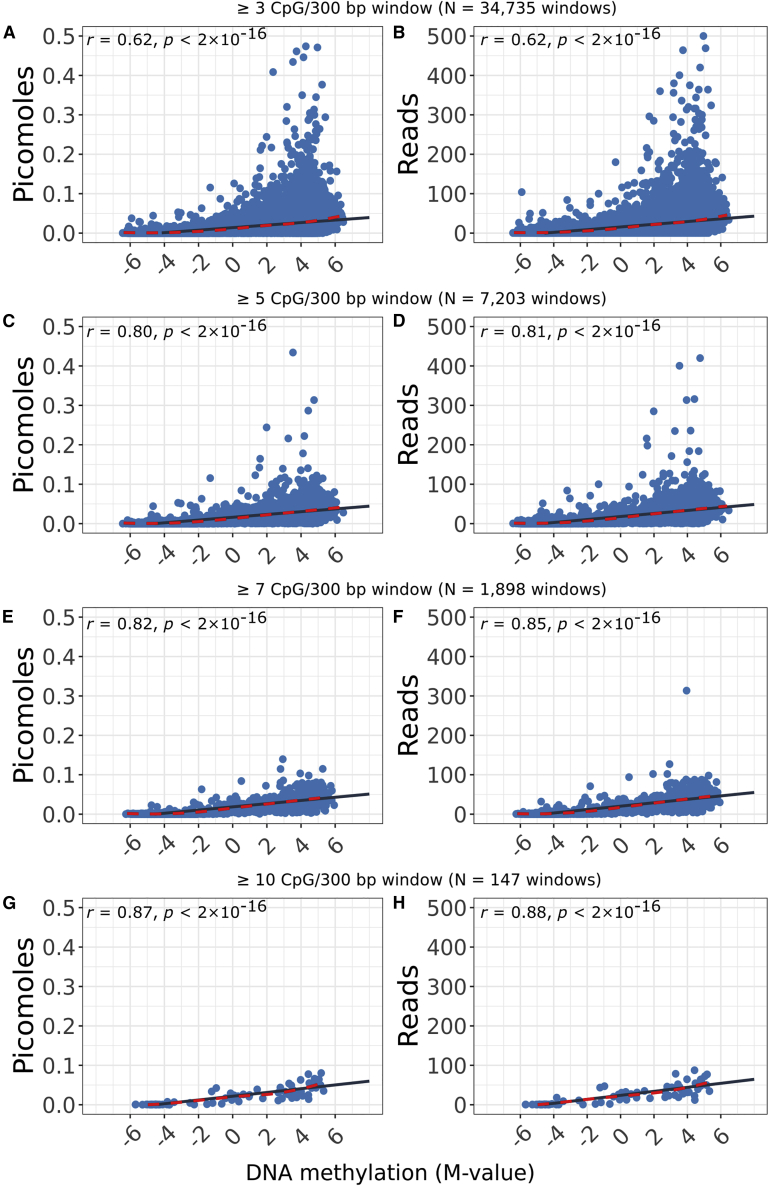
Correlation of two measurements of fragment methylation by cfMeDIP and EPIC array M-value for 300 bp genomic windows (A, C, E, and G) Molar amount calculated from HCT116 samples correlated to EPIC array M-values. (B, D, F, and H) Read counts calculated from the same samples, ignoring the spike-in controls. (A and B) 37,714 windows with ≥3 CpG probes represented on the EPIC array. (C and D) 7,975 windows with ≥5 CpG probes represented on the EPIC array. (E and F) 2,066 windows with ≥7 CpG probes represented on the EPIC array. (G and H) 158 windows with ≥10 CpG probes represented on the EPIC array. Solid black line, linear model of best fit; dashed red line, loess (Cleveland 1979) local regression.

We compared molar amount with M-values from the EPIC array (Figure 1A, right). We used our generalized linear model to calculate the molar amount of cfMeDIP-seq methylated DNA fragments for each 300 bp genomic window. Molar amount significantly correlated with array M-values over 300 bp in our HCT116 genomic DNA samples (r ≥ 0.62; Figures 4A, 4C, 4E, and 4G). Correlation increased when we restricted analyses to 300 bp windows with ≥5 CpG probes on the array (r = 0.82; Figure 4C). This reflected cfMeDIP-seq’s preference for methylated, CpG-dense reads.

We compared the current standard of read counts with M-values (Figures 4B, 4D, 4F, and 4H). Molar amount correlated with M-values similarly to read counts, but provided the advantage of absolute quantification.

### Spike-in controls accurately predict molar amount

**Figure 5:**
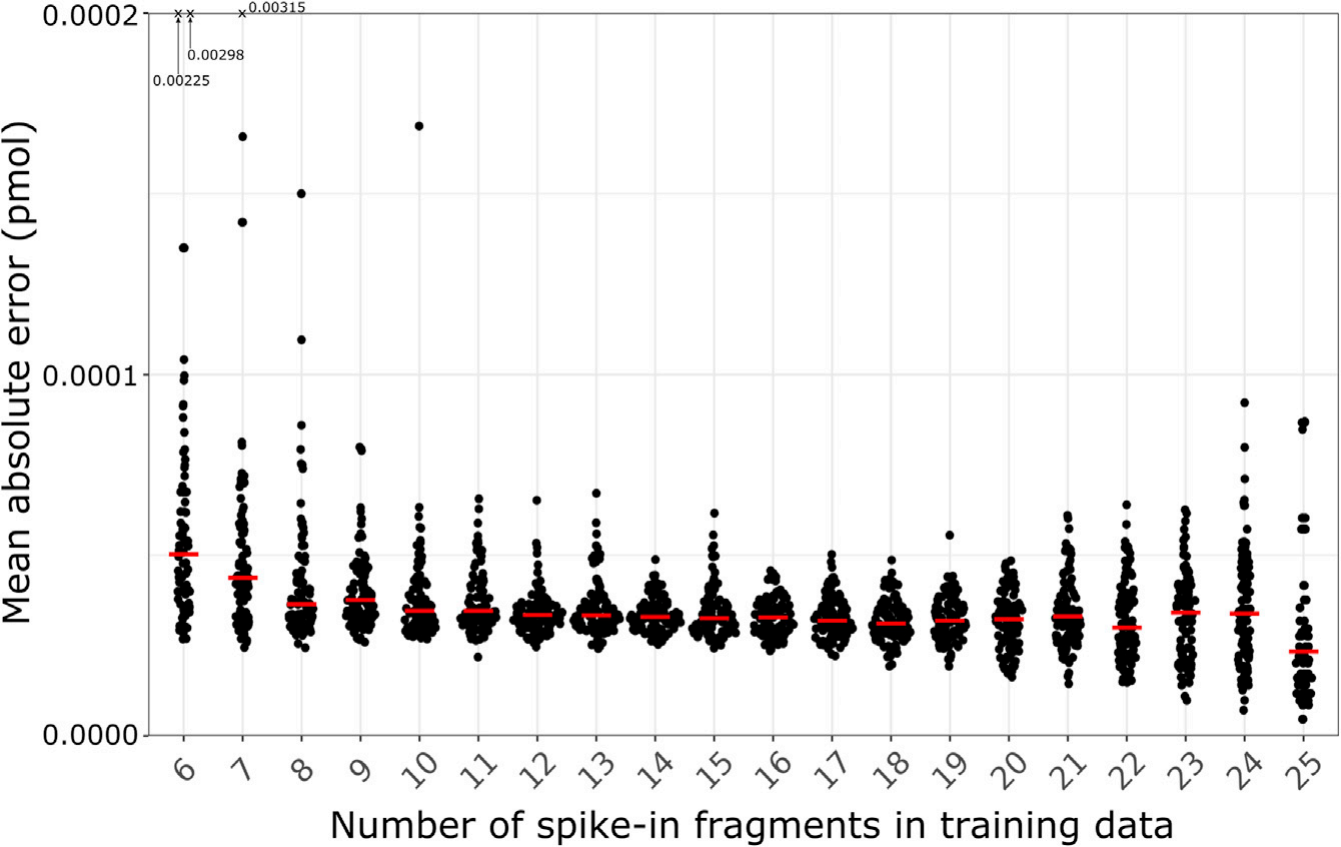
Mean absolute error between known molar amount and predicted molar amount in test data consisting of held-out spike-ins not used for training For each number of spike-in fragments between 6 and 25 inclusive, we 100 times randomly selected that number of spike-ins as training data. We used the remaining spike-ins as test data. Each point shows the mean absolute error over all the test spike-ins for that iteration. The vertical limits of the plot include at least 98/100 iterations in every case. We denote outliers for 6 or 7 training spike-ins with a cross at the top of the plot, labeled with the mean absolute error for that case. Red line denotes median mean absolute error. See also Table S6.

To assess the accuracy of models built on spike-in controls, we trained models using only some spike-ins as training data, testing on the held-out spike-ins. The mean absolute error between known molar amount and predicted molar amount was ≤0.002 pmol in every case. Adding more spike-in controls to the training data further increased model accuracy (Figure 5 and Table S4). While the largest training sets had the lowest median mean absolute error, they had larger variance in mean absolute error over 100 iterations. The increased variance likely derived from the increased noise from using smaller test sets, down to a single test spike-in used for the case with 25 training spike-ins.

### Spike-in controls significantly mitigate batch effects

To determine whether our spike-in controls mitigate batch effects for a clinical test, we provided aliquots of cfDNA samples from five AML patients to three different labs (Figure 1B). Each lab performed cfMeDIP-seq on each of the five samples with slight variations. We preprocessed each batch as described above. This yielded three models (Table S5).

**Figure 6:**
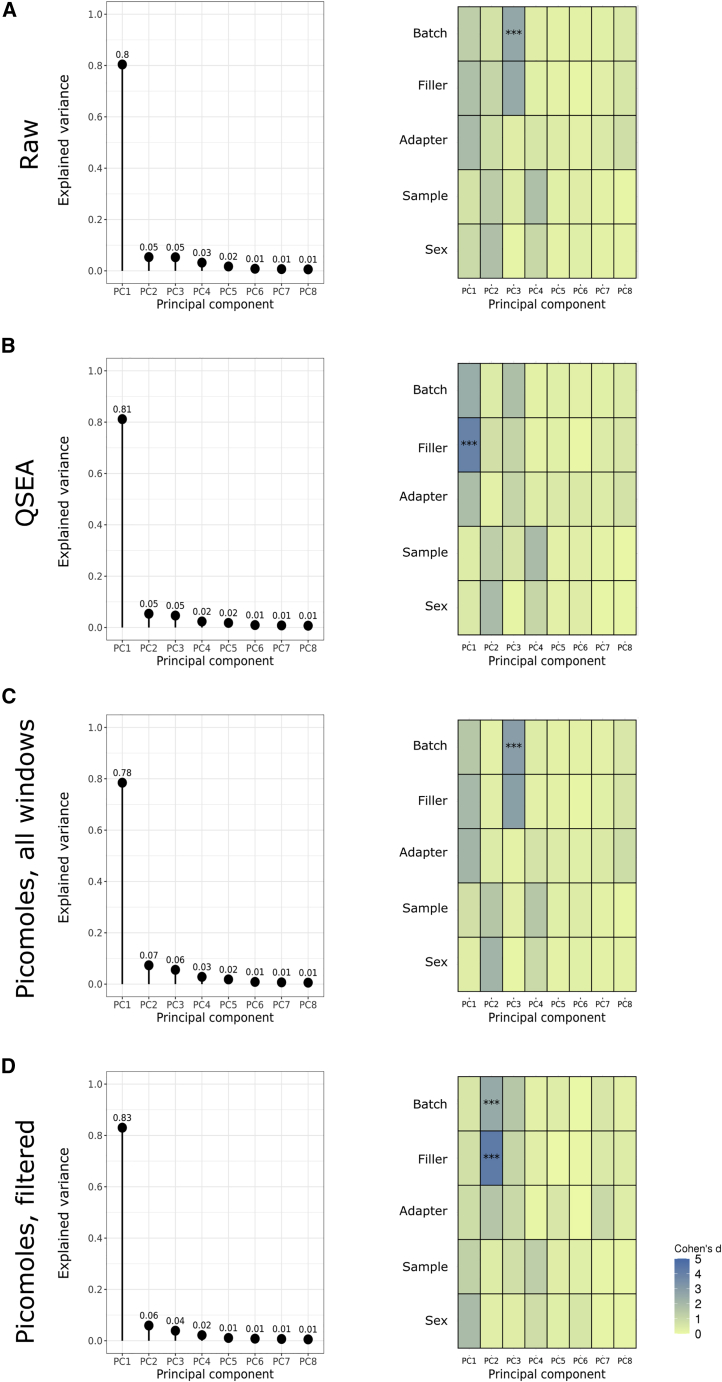
Principal component analyses of cfMeDIP results normalized through four different strategies, and associations with experimental variables (Left) Proportion of the variance explained by each principal component. (Right) Association between known variables, both technical and clinical, and principal component. Cohen’s *d* is an effect size of standardized means between variable. ∗∗∗p < 0.001. (A) Raw read counts without any normalization. (B) Read counts normalized using QSEA. (C) Data normalized using spike-in controls. (D) Data normalized using spike-in controls and removing regions in UCSC simple repeats, in the ENCODE blacklist, and with Umap k100 multi-read mappability scores ≤0.5. See also Tables S3, S4, and S5.

We performed principal component analysis (PCA) to assess whether any batch variables drive any of the top principal components. For raw read count data without spike-in controls, principal component 1 explains 80% of the variance and showed DNA methylation changes non-significantly associated with processing batch, filler (methylated or unmethylated), and adapter. For the raw reads, principal component 3, explaining 5% of the variance, was significantly associated with batch (Figure 6A).

Quantitative sequencing enrichment analysis (QSEA) normalization increased the variance explained by principal component 1 to 81%. After QSEA normalization, principal component 1 became significantly associated with filler. Principal component 1 was also associated non-significantly with batch and adapter (Figure 6B). The correlation of principal component 1 with filler DNA only appeared after QSEA normalization, suggesting that QSEA may have introduced spurious variance into the data.

Using spike-in controls reduced the variance explained by principal component 1 to 78%, which shows a non-significant effect size change in DNA methylation associated with batch variables. Similar to the raw data, we still see association with the batch variable in principal component 3, explaining 6% of the variance (Figure 6C).

Using our suggested filtering, which includes removing simple repeats, ENCODE blacklist regions, and regions with low mappability, improves variance associated with batch variables in the data. Upon removing these regions, batch and filler was associated with principal component 2, explaining 6% of the variance. These variables were not confounded with the known non-technical, biological variables—sample and sex—that one would want to associate with the top principal components. Principal component 1, explaining 83% of the variance, was associated with sample and sex more than other variables, though not significantly (Figure 6D). These changes in DNA methylation associated with biological variable were absent in principal component 1 of either the raw data (Figure 6A) or the QSEA data (Figure 6B). Removing the confounding of these changes with batch variables aids their use for assessing biological variance of interest.

We further investigated principal component 2 using spike-in controls and filtering. Examining the top 10% of windows (N = 258,254) driving the explained variance, 71% matched to RepeatMasker (Smit et al., 2010) repetitive elements. Of the mapped repetitive elements, 43% mapped to Alu elements. Batch effects associated with repetitive elements suggest inconsistent or inaccurate measures of DNA methylation at these regions.

To examine whether spike-in controls reduce technical variance, we performed sample-sample correlations in the raw read count data and predicted molar amount using spike-in controls. For each of the five AML samples, we compared the Lab B and Lab C data against Lab A. This generated 10 correlations for raw read count data and a paired 10 correlations for molar amount. We expect technical replicates to have correlations closer to 1. In all 10 of the technical replicate pairings examined, the molar amounts performed better than the raw read count data (Table S6).

## Discussion

The data presented here establish the validity of using our synthetic spike-in control DNA to absolutely quantify cfDNA in cfMeDIP-seq experiments. We showed that technical bias exists in the cfMeDIP-seq data and that the use of our spike-in controls helps mitigate these biases. For example, Lab C used unmethylated lambda filler DNA, but the spike-in controls successfully mitigated the effect of this change. While using spike-in controls significantly mitigates batch effects compared with QSEA, the variance in the data explained by batch variables remains similar between spike-in controls and raw data. Major advantages of using spike-in controls over raw data include the ability to adjust for fragment length, G + C content, and CpG fraction, and to absolutely quantify cfDNA. Additionally, one can use the signal from unmethylated spike-in control reads to calculate methylation specificity for each sample. To reduce technical artifacts, one must remove problematic genomic regions prior to analysis.

Despite removing problematic genomic regions, some remaining regions had much higher predicted molar amount than all other genomic regions of a given standard deviation or mappability score. The high molar amount genomic regions consisted mostly of repetitive elements, predominantly short interspersed nuclear elements (SINEs) (Table 2). While these regions had high CpG density, our model already adjusted for CpG fraction. This made CpG density an unlikely driver of high molar amount.

The two highest molar amount genomic regions from our HCT116 colon cancer cells overlap *MIER2*, part of the MIER gene family, associated with suppression of colorectal cancer (Peng et al., 2017). This shows the utility of our method to reveal important pathways for the gene regulation of the sample examined by a liquid biopsy.

Out of the top five genomic regions we followed up, four had high expression in the testis (two regions overlapping *MIER2* and one region overlapping each of *RNF126 and EXOC2*) and one had high expression in the ovary (*PRPF4B*). Several cancers exhibit high expression of genes associated with cell lines and low methylation in these genes’ promoters (Kutilin 2020; Pfeifer 2018; Janson et al., 2017).

The chr19:343,501–343,800 window resided within the first intron of *MIER2*, overlapping the EH38E1929888 candidate *cis*-regulatory element from ENCODE SCREEN (Moore et al., 2020). This element has a proximal enhancer-like signature in other cell types, but not in HCT116. *HCN2* and *RNF126* windows also overlapped high-density CpG islands. The other *MIER2* window at chr19:308,701–309,000 contained a short coding exon and flanking intronic sequence on both sides. Both *MIER2* windows also overlapped high-density CpG islands.

We may see over-representation of high molar amount in some repetitive regions owing to the characteristic hypermethylation of these regions (Deininger 2011). Had we not removed many repetitive regions when removing regions listed in the ENCODE blacklist and regions with low mappability, we may have observed more genomic regions with predicted high molar amount. Regions with high molar amount that map to repetitive elements contain many extra copies not present in the reference genome. These regions would appear uniquely mappable, and thus were not removed by our previous filtering steps.

HCT116 likely has problematic genomic regions not found in the ENCODE blacklist. This arises from the relative dearth of ENCODE data available for HCT116 in the blacklist generation process, compared with cell types in the ENCODE tier 1 and tier 2 categories.

Depending on the experimental question, some may choose to go beyond our filtering recommendations and remove all repetitive elements, such as all long interspersed nuclear elements (LINEs) and all SINEs. Given that, after filtering, only 11 genomic windows had molar amount ≥0.25 pmol, removing all repetitive elements would not affect results drastically.

Both molar amount in picomoles and read counts correlated with M-values. The strength of this correlation varied with the number of CpGs represented on the EPIC array for a genomic window. We expect the increase in correlation with more CpGs as cfMeDIP-seq preferentially enriches for hypermethylated regions and picks up fewer fragments in CpG-sparse regions. Additionally, if the array has fewer probes representing a 300 bp genomic region than that region has CpGs, it may poorly represent DNA methylation for the entire 300 bp.

To facilitate the use of our spike-in controls, we have created an R package, spiky, to help process data generated from cfMeDIP-seq experiments that include the spike-in controls. This package trains the Gaussian generalized linear model and predicts molar amount in picomoles on user data. The spiky package is available on Bioconductor (Huber et al., 2015; https://bioconductor.org/packages/spiky) and GitHub (https://github.com/trichelab/spiky).

Incorporating these spike-in controls in future cfMeDIP-seq experiments will adjust for technical biases and mitigate batch effects, improving cfMeDIP-seq data overall.

### Limitations of the study

The spike-in controls presented here allow absolute quantification of cfDNA, but the limited number of controls focus on a specific set of DNA fragment properties. Spike-in controls encompassing a larger number of fragment lengths, CpG fractions, and G + C content will further improve absolute quantification.

Using both unmethylated and methylated spike-in controls can estimate methylated DNA enrichment efficiency, but this applies to an entire experiment or sample. We still cannot tell whether any given fragment remaining after cfMeDIP is methylated or unmethylated, although we know that almost all of them are.

## STAR★Methods

### Key resources table

**Table:** 

REAGENT or RESOURCE	SOURCE	IDENTIFIER
**Antibodies**		

5-methylcytosine, clone 33D3	Diagenode, Denville, NJ, USA	Cat# C1500081

**Biological samples**		

AML sample 6	Leukemia Tissue Bank, Princess Margaret Cancer Centre, Toronto, ON, Canada	150279
AML sample 7	Leukemia Tissue Bank, Princess Margaret Cancer Centre, Toronto, ON, Canada	151050
AML sample 8	Leukemia Tissue Bank, Princess Margaret Cancer Centre, Toronto, ON, Canada	160537
AML sample 9	Leukemia Tissue Bank, Princess Margaret Cancer Centre, Toronto, ON, Canada	160326
AML sample 10	Leukemia Tissue Bank, Princess Margaret Cancer Centre, Toronto, ON, Canada	150197

**Chemicals**, **peptides**, **and recombinant proteins**		

High-Fidelity 2X Master Mix	New England Biolabs, Ipswich, MA, USA	Cat# M0492L
QIAquick PCR Purification Ki	Qiagen, Hilden, Germany	Cat# 28104
*M*.*SssI* CpG methyltransferase	Thermo Fisher Scientific, Waltham, MA, USA	Cat# EM0821
MinElute PCR Purification Kit	Qiagen, Hilden, Germany	Cat# 28004
HpyCH4IV	New England Biolabs, Ipswitch, MA, USA	Cat# R0619S
HpaII	New England Biolabs, Ipswitch, MA, USA	Cat# R171S
AfeI	New England Biolabs, Ipswitch, MA, USA	Cat# R0652S

**Experimental models**: **Cell lines**		

Mycoplasma-free HCT116 genomic DNA	American Type Culture Collection, Manassas, VA, USA	CCL-247; RRID: CVCL_0291

**Oligonucleotides**		

Synthetic spike-in control oligonucleotides, see Table S1	This paper	N/A
Ultramer DNA Oligonucleotides	IDT, Coralville, IA, USA	N/A
gBlocks Gene Fragments	IDT, Coralville, IA, USA	N/A
xGen Stubby Adapter and UDI primer pairs	IDT, Coralville, IA, USA	Cat# 10005924

**Deposited data**		

Raw cell line data	This paper	GEO: GSE166259
Raw AML patient data	This paper	EGA: EGAS00001005069
Processed AML patient data	This paper	GEO: GSE166259
Human reference genome GRCh38/hg38	Genome Reference Consortium	RefSeq: GCF_000001405.39

**Software and algorithms**		

GENCODE v.33	Frankish et al. (2019)	https://www.gencodegenes.org/human/release_33.html
RepeatMasker v.3.0	Institute for Systems Biology, Seattle, WA, USA	https://www.repeatmasker.org/
GenRGenS	Ponty et al. (2006)	https://www.lri.fr/genrgens/
RNAStructure v 6.2	Xu and Mathews (2016)	https://rna.urmc.rochester.edu/RNAstructure.html
Fastp v.0.11.5	Chen et al. (2018)	https://github.com/OpenGene/fastp
Bowtie2 v.2.3.5	Langmead and Salzberg (2012)	http://bowtie-bio.sourceforge.net/bowtie2/index.shtml
UMI-tools version 1.0.0	Smith et al. (2017)	https://github.com/CGATOxford/UMI-tools
Samtools v. 0.10.2	Li et al. (2009)	https://github.com/samtools/samtools
R v. 3.4.1	R Core Team (2013)	https://cran.r-project.org/bin/windows/base/old/3.4.1/
Bedtools v.2.29.2	Quinlan and Hall (2010)	https://github.com/arq5x/bedtools2
Sesame v. 1.8.2	Zhou et al. (2018)	https://github.com/zwdzwd/sesame
QSEA v. 1.16.0	Lienhard et al. (2017)	https://github.com/MatthiasLienhard/qsea
compute.es v.0.2.5	Comprehensive R Archive Network (CRAN)	https://cran.r-project.org/package=compute.es
spiky	This paper	https://github.com/trichelab/spiky; https://doi.org/10.18129/B9.bioc.spiky
All code pertaining to this paper	This paper	https://github.com/hoffmangroup/2020spikein; https://doi.org/10.5281/zenodo.4683791

### Resource availability

#### Lead contact

Further information and requests for resources and reagents should be directed to and will be fulfilled by the lead contact, Michael M. Hoffman (michael.hoffman@utoronto.ca).

#### Materials availability

This study did not generate new unique reagents. One can order the spike-in DNA fragments described here from commercial providers.

### Experimental model and subject details

#### HCT116 cell line

We purchased mycoplasma-free HCT116 colon cancer cells (American Type Culture Collection, Manassas, VA, USA). The supplier authenticated the cells through short tandem repeat analysis (Cell Line Authentication Service, American Type Culture Collection, Manassas, VA, USA) and Sanger sequencing. HCT116 cells are male. We extracted DNA from these cells directly from the supplier without culturing.

#### AML clinical samples

We obtained deidentified plasma samples, previously included in Shen et al. (2018), from 5 AML adult patients (Leukemia Tissue Bank, Princess Margaret Cancer Centre, University Health Network, Toronto, ON, CA). The patients gave informed consent following approval by the University Health Network Research Ethics Board (01-0573). The samples consist of sample 6 (female, age 62), sample 7 (female, age 79), sample 8 (male, age 78), sample 9 (male, age 67), and sample 10 (female, age 45).

Gender information about the patients was not recorded. Analysis comparing males and females lies beyond the scope of this work, as it focuses on technical variation rather than biological characterization.

### Method details

#### Designing synthetic DNA spike-in controls

We used public paired-end, whole genome cfDNA sequence data to assess typical cfDNA fragment properties including fragment length, G+C content, and the number of CpG dinucleotides (Mouliere et al., 2018). We considered the number of CpGs as a fraction of fragment length.

From the observed distribution of cfDNA fragment properties, we set the following spike-in fragment parameters:

• 3 fragment lengths: 80 bp, 160 bp, and 320 bp
• 3 G + C contents: 35%, 50%, and 65%
• 3 CpG fractions: 1/80 bp, 1/40 bp, and 1/20 bp

These parameters generate 27 fragment combinations (3 fragment lengths × 3 G + C contents × 3 CpG fractions = 27).

We set the CpG fraction parameters so that every fragment length would have an integer number of CpGs. For example, the 80 bp fragments have 1, 2, or 4 CpG dinucleotides, and the 160 bp fragments have 2, 4, and 8 CpG dinucleotides.

We used GenRGenS version 2.0 (Ponty et al., 2006) to construct 27 different first-order Markov models that generate sequences with the desired parameters. For each Markov model, we generated numerous sequences. We then identified those sequences that fulfilled two criteria: (1) no alignment to human genome and (2) no potential secondary structures.

Using blastn (Altschul et al., 1997), we searched for alignment of the generated sequences to the human reference genome (GRCh38/hg38) (Schneider et al., 2017). We ensured no alignment of each synthetic sequence to the genome. To do this, we selected the sequences with the highest E-values in each search, checking that none of them had an alignment bit score greater than 50.

We used UNAFold software (Owczarzy et al., 2008) (Integrated DNA Technologies (IDT), Coralville, IA, USA) to check for secondary DNA structure for 80 bp and 160 bp fragments. For 320 bp fragments, we used RNAstructure version 6.2 (Xu and Mathews 2016) to check for secondary DNA structures.

We attempted to pick two sequences from those fulfilling our criteria for each of the 27 Markov models. This represented one sequence for a methylated fragment, and one for an unmethylated fragment. If possible, this would have produced 54 desired spike-in control sequences. The generated 320 bp fragments with 65% G + C content, however, had excessive amounts of secondary structure, which would make synthesis difficult and might have systematic effects on the cfMeDIP process. To retain a total of 18 generated spike-in control sequences for 320 bp fragments, we designed an additional alternative set of fragments with different sequences for each of 3 combinations of G + C content and CpG fraction: (1) 35% G + C content, 1/80 bp CpG fraction; (2) 50% G + C content, 1/40 bp CpG fraction; and (3) 50% G + C content, 1/20 bp CpG fraction.

#### Synthetic fragment preparation

We acquired synthetic fragments for the spike-in control sequences from a commercial service (IDT, Coralville, IA, USA). For the 80 bp and 160 bp fragments we used 4 nmol Ultramer DNA Oligonucleotides. For the 320 bp fragments we used gBlocks Gene Fragments, obtaining 250 ng of each designed fragment. The two 160 bp fragments with 35% G + C content and 1/20 bp CpG fraction failed commercial design procedures, leaving us with 52 spike-in control fragments (Table S1).

We amplified the synthetic fragments by PCR. We used the High-Fidelity 2X Master Mix (New England Biolabs, Ipswich, MA, USA, Cat #M0492L) and the fragments’ optimal annealing temperatures (Table S1). We purified amplified fragments using the QIAquick PCR Purification Kit (Qiagen, Hilden, Germany, Cat #28104). We determined concentration of each synthetic fragment via NanoDrop (Thermo Fisher Scientific, Waltham, MA, USA).

For each fragment designed for methylation, we took 1 μg of synthetic DNA fragment, and methylated using *M*.*SssI* CpG methyltransferase (Thermo Fisher Scientific, Waltham, MA, USA, Cat #EM0821). We incubated the methylation reaction at 37 °C for 30 min, then 65 °C for 20 min. We purified the methylated product using the MinElute PCR Purification Kit (Qiagen, Hilden, Germany, Cat #28004). To verify methylation, we digested the original PCR amplicon and the methylated PCR amplicon with either HpyCH4IV, HpaII or AfeI restriction enzyme, depending on the cut sites each fragment contained (Table S1). We considered methylation successful when, after restriction digest, the PCR amplicon had a single band when run on a 2% agarose electrophoresis gel. Then, using the known relative molecular mass of each synthetic fragment, we determined its molar amount using the Qubit dsDNA HS Assay Kit and a Qubit 2.0 Fluorimeter (Thermo Fisher Scientific, Waltham, MA, USA).

#### Assessing technical bias

To assess the performance and any potential biases of synthetic fragments as spike-in controls, we performed cfMeDIP-seq using solely the spike-in control DNA pools as the input. The input pool consisted of 9.99 ng synthetic spike-in DNA, with equimolar amounts of each fragment size within each fragment size pool, and equimolar amounts of each methylation status (Table S1).

We performed cfMeDIP-seq as previously described (Shen et al., 2019) with slight modifications, detailed here. To account for PCR amplification bias, we used xGen Stubby Adapter and unique dual indexing (UDI) primer pairs (IDT, Coralville, IA, USA, Cat #10005924). We performed adapter ligation overnight at 4 °C, adjusting final adapter concentration to 0.09 μmol by dilution.

For each sample, we saved 10% of the DNA denaturation product, prior to incubation with the antibody, as input (Figure 1A, Input 1A and Input 2A). For each sample, we amplified both input and outputs followed by purification and dual size selection using AMPure XP beads (Beckman Coulter, Brea, CA, USA) selecting for fragments between 200 bp–500 bp, reflecting the addition of adapter to the original DNA fragments. These post-ligation fragment sizes reflect an original fragment length between 80 bp–380 bp. Samples underwent sequencing (Princess Margaret Genomics Centre, Toronto, ON, Canada) using the 300-cycle MiSeq Reagent Kit V2 on a MiSeq 2.0 Nano flowcell (Illumina, San Diego, CA, USA), paired-end 2 × 150 bp, 1 million reads per flowcell (Figure 1A).

#### Optimizing synthetic DNA amount

We determined the optimal amount of spike-in control DNA needed per experiment by adding varying amounts of spike-in controls to sheared HCT116 genomic DNA. Following this addition with cfMeDIP-seq allowed controlled assessment of how our spike-in controls behave in the context of a biological experiment. Optimizing the amount of spike-in control DNA added to an experiment avoids using a large portion of the sequencing reads on spike-in fragments, saving most reads for the biological sample. We sheared the HCT116 genomic DNA using an LE220 ultrasonicator (Covaris, Woburn, MA, USA). Using AMPure XP beads (Beckman Coulter, Brea, CA, USA), we size selected to ∼150 bp in length to mimic cfDNA input. We created 3 replicate samples of sheared HCT116 cfDNA mimic with masses of synthetic spike-in control DNA of 0.1, 0.05, and 0.01 ng. We performed the cfMeDIP-seq experiment as previously described (Shen et al., 2019). Samples underwent sequencing (Princess Margaret Genomics Centre, Toronto, ON, Canada) on a NovaSeq 6000 (Illumina, San Diego, CA, USA), paired-end 2 × 100 bp, 60 million paired-end reads per sample (Figure 1A).

#### Bioinformatic preprocessing

We performed the same bioinformatic preprocessing on all samples from all experiments. We trimmed adapters using fastp version 0.11.5 (Chen et al., 2018) --umi --umi_loc=per_read --umi_len=5 --adapter_sequence=AATGATACGGCGACCACCGAGATCTACACATATGCGCACACTCTTTCCCTACACGAC --adapter_sequence_r2=CAAGCAGAAGACGGCATACGAGATACGATCAGGTGACTGGAGTTCAGACGTGT. We removed reads with a Phred score (Ewing and Green 1998) <40, the default of Bowtie2, and reads with mapping quality <20. We aligned reads to the sequences of our designed fragments using Bowtie2 (Langmead and Salzberg 2012) version 2.3.5 bowtie2 --local --minins 80 --maxins 320, writing unaligned reads to a separate file. We subsequently aligned the previous unaligned reads to our synthetic DNA to the human reference genome (GRCh38/hg38) (Schneider et al., 2017). In every sample, over 98% of reads aligned to either spike-in control sequences or to the human genome. We discarded read pairs when at least one read in the pair did not align or had low quality, defined as Phred score (Ewing and Green 1998) <40 or a mapping quality score <20. We collapsed fragments with the same UMI files counting them as one fragment using UMI-tools version 1.0.0 (Smith et al., 2017). We used samtools (Li et al., 2009) version 0.10.2 to convert sequence alignment/map (SAM) files to binary alignment/map (BAM) files (Li et al., 2009).

### Quantification and statistical analysis

#### Absolute quantification from spike-in control data

We created a Gaussian generalized linear model to predict molar amount from deduplicated spike-in control read counts based on UMI consensus sequence, G + C content, CpG fraction, and fragment length. Using this method, we can absolutely quantify cfDNA. To do this, we used the stats package in R (R Core Team 2013) version 3.4.1. Our model estimates molar amount η for each DNA fragment present in the original sample using regression coefficients β learned for each experiment. For each fragment, this model directly includes read count $x_{\text{reads}}$, fragment length $x_{\text{len}}$, G + C content $x_{\text{GC}}$, and CpG fraction $x_{CpG\;fraction}$. The final model estimates the molar amount η,

$$\eta =\beta_{0}+\beta_{\text{reads}}x_{\text{reads}}+\beta_{\text{len}}x_{\text{len}}+\beta_{\text{GC}}x_{\text{GC}}+\beta_{CpG\;fraction}\sqrt[{3}]{x_{CpG\;fraction}}\text{.}$$

to reduce the left skew of CpG fraction, we used a cube root transformation.

To calculate the proportion of a given fragment that overlapped with the defined 300 bp windows, we used bedtools version 2.29.2 intersect (Quinlan and Hall 2010). We calculated an adjusted molar amount $\eta^{\prime }$ to only consider the portion of the window each fragment overlapped. For this calculation, we multiplied the molar amount η by the length of the overlap between the fragment and the genomic window ℓ∈ [1 bp, 300 bp], divided by the window size $\ell^{*}=$ 300 bp:

$$\eta^{\prime }=\frac{\ell }{\ell^{*}}\eta \text{.}$$

#### Identifying regions to remove during filtering

To assess multimapping reads that might influence the molar amount estimate, we used Umap (Karimzadeh et al., 2018) multi-read mappability scores. We used k100 mappability scores, representing the largest read lengths available. We annotated each 300 bp window with its minimum mappability score. We assessed the relationship between molar amount and mappability scores.

We calculated standard deviation of molar amount between the two replicate samples for which we spiked 0.01 ng of synthetic DNA into 10 ng of HCT116 genomic DNA. We assessed the relationship between standard deviation of molar amount between replicates, excluding 1,0 70,387 simple repeat regions (Karolchik et al., 2004), 239,461 regions listed in the ENCODE Project (ENCODE Project Consortium 2012) blacklist (Amemiya et al., 2019), 549,876 regions with mappability score ≤0.5, and 906 regions with standard deviation ≥0.05. This left 4,446,375 genomic windows in the analyses.

#### Correlation between picomoles and M-values

We removed simple repeat regions (Karolchik et al., 2004), regions listed in the ENCODE blacklist (Amemiya et al., 2019), regions with Umap k100-multi-read mappability ≤0.5, and regions with standard deviation of molar amount ≥0.05. As described above, we estimated molar amount, using a generalized linear model $\left(r^{2}=0.93\right)$. We prioritized models that performed better on 160 bp fragments, as we physically size selected for these fragments.

To compare molar amount to another complimentary measure of DNA methylation, we had genomic DNA from the cell line HCT116 profiled (Princess Margaret Genomics Centre, Toronto, ON, Canada) using the Infinium MethylationEPIC BeadChip array (Illumina, San Diego, CA, USA). We prepared these samples as technical replicates of the HCT116 genomic DNA that we later spiked with 0.01 ng spike-in control. We normalized and preprocessed array data using sesame (Zhou et al., 2018) version 1.8.2. We annotated CpGs on the array to our 300 bp genomic windows. When >1 CpG probe annotated to a window, we calculated the mean probe M-values across the window.

We assessed correlation between array M-values and picomoles, and between array M-values and read counts. We compared to M-values rather than β values because β values have high heteroscedasticity (Du et al., 2010). As cfMeDIP-seq preferentially enriches for highly methylated regions, we hypothesized that regions for which the array has more CpG probes would correlate to both molar amount and read counts better than regions with less CpG coverage. As such, we assessed the correlation independently at windows containing ≥3, ≥5, ≥7, and ≥10 CpG probes.

#### Examining accuracy of predicted molar amount

To determine how well our spike-in controls predict molar amount, we trained Gaussian generalized linear models using only some spike-ins as training data, and held-out spike-ins as test data. We performed this analysis on AML data from Lab A. We chose Lab A as this lab adhered most closely to the previously published protocol (Shen et al., 2018).

We parameterized generalized linear models using training sets containing at least 6 and at most 25 spike-in controls. Since the model had 5 terms, using a minimum of 6 spike-ins meant we always had more training data points than the number of terms in the model. This avoided likely inaccurate rank-deficient fits of the model. Then, we predicted the remaining spike-in controls, which had known molar amount, as a test set. For each number of spike-in controls for training, we randomly selected 100 different training sets. For each of these 100 iterations, we calculated the mean absolute error between the known molar amount and predicted molar amount.

#### Examining consistency across experimental batches

To experimentally introduce batch effects, we provided a sample of 10 ng of cfDNA obtained from peripheral blood plasma of 5 AML patients containing 0.01 ng of our spike-in controls to 3 independent labs (Figure 1B). Blood was collected at the time of diagnosis in EDTA tubes. Samples were spun and plasma frozen in Eppendorf tubes at −80 °C until use. As a blood cancer, leukemia generates a high amount of plasma cfDNA, allowing us to have sufficient cfDNA to divide into three technical replicates of 10 ng each.

Each lab performed the cfMeDIP-seq method described by Shen et al., (2019), with a variety of procedural modifications, detailed here. The changes emulated batch effects commonly found in publicly available data from different labs. Labs A and C used the same IDT xGen Duplex Seq adapters with 3 bp–4 bp UMI, as described above. Lab B used custom IDT adapters with 2 bp degenerate UMI, as previously described (Wang et al., 2019). For ligation of adapters, Labs A and B incubated at 4 °C for 16 h, while Lab B incubated at 20 °C for 2 h. Labs A and C used Antibody 1 (Diagenode, Denville, NJ, USA, Cat #C15200081-100, Lot #RD004, RRID: AB_2572207), while Lab B used Antibody 2 (Diagenode, Denville, NJ, USA, Cat #C15200081-100, Lot #RD001, RRID: AB_2572207). Lab A and B used 50% methylated lambda filler DNA and 50% unmethylated lambda filler DNA. Lab C used 100% unmethylated lambda filler DNA. The lambda filler tops up the sample to 100 ng so that each sample has the same amount of input DNA.

For amplifying the final library, the batches had different numbers of PCR cycles. Lab A ran 13–15 cycles, optimized per sample, Lab B ran 13 cycles, and Lab C ran 11 cycles. Lab A and B sequenced DNA with a NovaSeq 6000 (Illumina, San Diego, CA, USA), paired-end 2 × 100 bp. Lab C sequenced DNA on a NextSeq 550 (Illumina, San Diego, CA, USA), paired-end 2 × 75 bp. Lab A obtained 60 million reads per sample, Lab B obtained 100 million reads per sample, and Lab C obtained 85 million reads per sample.

We assessed whether spike-in controls mitigated batch effects on samples for which we calculated molar amount. To do this, we performed PCA on different quantification methods, which included raw read counts, our molar amount estimates, and the output of QSEA (Lienhard et al., 2017), the current standard processing pipeline for MeDIP-seq data. QSEA normalizes MeDIP-seq data without spike-in controls, taking into account fragment length, G + C content, and number of CpGs. Specifically, we examined the following four methods:

1. read counts only, without the use of spike-in controls
2. read counts preprocessed using QSEA version 1.16.0,
3. molar amount, without filtering
4. molar amount, with filtering for repeat regions, ENCODE blacklist, and regions with low mappability

To investigate whether known variables associated with any of the principal components, we performed two-way analysis of variance (ANOVA) between each principal component and each categorical variable. Categorical variables included batch, filler (methylated or unmethylated), adapter, samples, and sex (inferred by Y chromosome signal). We converted the resulting F-statistic to an effect size, Cohen’s d (Rice and Harris 2005), using the compute.es package (Del Re 2013) version 0.2.5 and R version 3.4.1. We adjusted p values for multiple test correction using the Holm-Bonferroni method (Holm 1979).

## Data Availability

• cfMeDIP-seq cell line data have been deposited at Gene Expression Omnibus (GEO; Edgar et al., 2002) and are publicly available as of the date of publication. The accession number is listed in the key resources table. • De-identified raw cfMeDIP-seq human AML data have been deposited at the European Genome-phenome Archive (EGA; Lappalainen et al., 2015), and the accession number is listed in the key resources table. They are available upon request if access is granted. To request access, contact the University Health Network Genomics Data Access Committee (dac@uhn.ca) following the instructions in the specified policy for processing data access requests (https://doi.org/10.5281/zenodo.4568265). In addition, processed data have been deposited at Gene Expression Omnibus (GEO) and are publicly available as of the date of publication. The accession number is listed in the key resources table. • The spiky package has been deposited at Bioconductor and is publicly available as of the date of publication. All other original code has been deposited at Zenodo and is publicly available as of the date of publication. Digital Object Identifiers (DOIs) are listed in the key resources table. The code is also available on GitHub. URLs are listed in the key resources table. • Any additional information required to reanalyze the data reported in this paper is available from the lead contact upon request.

## Supplemental information

Table S1. Spike-in control sequences, related to Table 1 and STAR Methods

Table S2. Read counts, related to Figure 2

Table S3. Raw read count data for spike-in controls used in AML samples, related to Figure 6

Table S4. Mean absolute errors from training with varying numbers of spike-ins, related to Figure 6

Table S5. Models and their regression coefficients, related to Figure 6

Table S6. Sample-sample correlation matrices for raw data and predicted molar amount, related to Figure 5
